# Comparative analysis of dermal and inhalation exposures to antineoplastic drugs among workers in the workplaces: a systematic review

**DOI:** 10.1186/s12889-024-21191-4

**Published:** 2025-05-15

**Authors:** Zahra Beigzadeh, Farideh Golbabaei, Fariborz Omidi, Seyed Jamaleddin Shahtaheri

**Affiliations:** 1 Department of Occupational Health, School of Public Health, Tehran University of Medical Sciences, Tehran, Iran; 2 Research Center for Environmental Determinants of Health, School of Public Health, Kermanshah University of Medical Sciences, Kermanshah, Iran

**Keywords:** Antineoplastic drugs, Occupational health, Dermal exposure, Inhalation exposure, Systematic review

## Abstract

**Objective:**

Occupational exposure to antineoplastic drugs presents significant health risks to workers, necessitating a comprehensive understanding of both dermal and inhalation exposures. This systematic review examines the relative significance of cutaneous versus inhalation exposure among professionals handling these potent medications.

**Study design:**

Systematic review.

**Methods:**

A systematic search using the PECO framework was conducted in PubMed, Scopus, and Web of Science, adhering to PRISMA guidelines. Data from surface and air sampling studies were collected and analyzed.

**Results:**

Ten studies met the inclusion criteria, assessing various antineoplastic drugs across different occupational settings. Surface contamination levels varied widely, with concentrations ranging from very low to high, whereas airborne monitoring consistently reported "Not Detectable" levels. Exposure levels were influenced by workplace practices, handling procedures, and the sensitivity of detection methods.

**Conclusions:**

This systematic review of ten studies on dermal and inhalation exposure to antineoplastic drugs in various occupational settings reveals significant variability in contamination levels. Tailored safety measures, including stringent protocols, decontamination procedures, and respiratory protection, are essential for workplace safety. The review highlights the importance of standardized safety protocols, considering the impact of workplace practices and detection method sensitivity. Additionally, it underscores the health risks associated with even low-level exposure, emphasizing the need for biological monitoring. Despite some limitations, this study offers valuable insights for enhancing the safety of staffs handling these potent drugs, guiding future research and policy development.

## Impact statement

This systematic review highlights the significant variability in cutaneous and inhalation exposures to antineoplastic medications among workers in various occupational settings, addressing a critical gap in occupational health. Our findings underscore the necessity of specialized safety measures, including strict protocols and appropriate dermal and respiratory protection, by demonstrating contamination levels across different environments. This study offers essential insights for safeguarding healthcare workers, informing future regulations, and emphasizing the need for standardized safety protocols. The global impact of our research is evident in its potential to shape occupational health practices and create safer working conditions for those handling hazardous pharmaceuticals.

## Introduction

Occupational exposure to antineoplastic drugs has become a major concern due to its potential negative impacts on the health and well-being of workers in various occupational settings. Antineoplastic drugs, widely used in chemotherapy, possess potent cytotoxic properties that effectively target and eliminate cancer cells. However, these same properties pose inherent risks to individuals involved in the production, administration, and disposal of these medications [1, 2]. Dermal and inhalation exposures are recognized as the primary routes through which workers may come into contact with these hazardous substances [3–5].

Dermal exposure occurs when antineoplastic drugs come into direct contact with the skin through spills, splashes, or contact with contaminated surfaces [6, 7]. This pathway is particularly concerning because these drugs can penetrate the skin barrier, enter the bloodstream, and potentially cause systemic absorption and subsequent health effects [8, 9]. Inhalation exposure, on the other hand, involves the inhalation of airborne particles generated during various processes, such as drug preparation, administration, and cleanup, resulting in suspended particles or aerosols [7, 10]. Inhaling these particles may lead to their accumulation in the respiratory system, enabling direct absorption into the bloodstream or causing localized respiratory effects [11].

Determining whether cutaneous exposure to antineoplastic drugs in the workplace exceeds inhalation exposure is critical. Although several studies have examined occupational exposure to these drugs, there is currently no comprehensive review comparing the relative levels of dermal and inhalation exposures. Understanding the primary routes of exposure is essential for effectively implementing preventive measures. Developing appropriate control strategies is crucial to safeguarding the health and well-being of professionals who handle these potentially harmful substances in various occupational settings.

The primary objective of this systematic review is to address this knowledge gap by consolidating recent research on the exposure of workers in diverse occupational settings to antineoplastic drugs via dermal and respiratory pathways. This study aims to determine the relative significance of dermal exposure compared to inhalation exposure through a thorough analysis of existing literature. Additionally, the research seeks to explore factors influencing variations in exposure levels, including job functions, working conditions, and the effectiveness of preventive measures.

## Materials and methods

### Study design

This systematic review addresses the research question: "Does dermal exposure to antineoplastic drugs among workers in the workplace exceed inhalation exposure?".

### Search strategy

The search strategy for this systematic review adhered to the Cochrane Collaboration's guidelines [12]. The study's aim was formulated using the PECO framework, encompassing Participants, Exposure, Comparison, and Outcome. Both the search and reporting processes followed the Preferred Reporting Items for Systematic Reviews and Meta-Analyses (PRISMA) guidelines [13] to ensure methodological rigor and transparency. The study's objective, framed within the PECO framework, specified the following:

- *Participants*: Workers (including pharmacists, nurses, physicians, operating room personnel, hospital staff such as transport and receiving personnel, janitors, laundry workers, and waste handlers) who handle or administer antineoplastic drugs in the workplace.
- *Exposure*: Dermal exposure to antineoplastic drugs.
- *Comparison*: Inhalation exposure to antineoplastic drugs.
- *Outcome*: The relative level of dermal exposure compared to inhalation exposure to antineoplastic drugs in the workplace.

Scopus, PubMed, and Web of Science databases were searched for English-language research articles published between 1977 and 2023. The following search strategies were employed:

- PUBMED: ("Antineoplastic Agents"[Mesh]) OR (Antineoplastic Drug[Title/Abstract]) OR (Chemotherapy Agent[Title/Abstract]) OR (Chemotherapy Drug[Title/Abstract]) OR (Cytotoxic drug[Title/Abstract]) OR (Antiblastic drug[Title/Abstract])) OR (Hazardous drug[Title/Abstract]) AND ("Occupational Exposure"[Mesh]) OR (Occupational Exposure[Title/Abstract])) OR ("Inhalation Exposure"[Mesh]) OR (Inhalation Exposure[Title/Abstract])) OR (dermal exposure)) OR (skin exposure[Title/Abstract]) AND ("Workplace"[Mesh]) OR (Workplace[Title/Abstract]) OR (occupation*[Title/Abstract]) OR (Worksite[Title/Abstract])
- SCOPUS: ( TITLE-ABS-KEY ( "Antineoplastic Agent") OR TITLE-ABS-KEY ( "Antineoplastic Drug") OR TITLE-ABS-KEY ( "Chemotherapy Agent") OR TITLE-ABS-KEY ( "Chemotherapy Drug") OR TITLE-ABS-KEY ( "Cytotoxic drug") OR TITLE-ABS-KEY ( "Antiblastic drug") OR TITLE-ABS-KEY ( "Hazardous drug") AND ( TITLE-ABS-KEY ( "Occupational Exposure") OR TITLE-ABS-KEY ( "Inhalation Exposure") OR TITLE-ABS-KEY ( "dermal exposure") OR TITLE-ABS-KEY ( "skin exposure")) AND ( TITLE-ABS-KEY ( workplace) OR TITLE-ABS-KEY ( occupation*) OR TITLE-ABS-KEY ( worksite)
- Web of Science: (TS = ("Antineoplastic Agent" OR "Antineoplastic Drug" OR "Chemotherapy Agent" OR "Chemotherapy Drug" OR "Cytotoxic drug" OR "Antiblastic drug" OR "Hazardous drug") AND (TS = ("Occupational Exposure" OR "Inhalation Exposure" OR "dermal exposure" OR "skin exposure") AND (TS = (workplace OR occupation* OR worksite))

### Selection of studies

EndNote 20 (Thomson Reuters, Toronto, Canada) was used to collect and manage the studies from the three databases. Duplicates were identified and removed using EndNote 20's automated functions, with manual confirmation to ensure the retention of only one record per article. The titles, abstracts, and full texts of the articles were then screened in sequence.

### Inclusion and exclusion criteria

Inclusion Criteria:

- Observational studies (e.g., cross-sectional, cohort) assessing workers' dermal and inhalation exposure to antineoplastic drugs in the workplace.
- Studies published in English.
- Studies reporting quantitative data on both dermal and inhalation exposure to antineoplastic drugs.

Exclusion Criteria:

- Studies not conducted in a workplace setting (e.g., simulations, controlled experiments).
- Studies that examined only dermal or inhalation exposure, not both.
- Studies reporting occupational contact with non-antineoplastic chemical substances.
- Studies not available in English.
- Studies lacking sufficient information about the population or exposure characteristics.
- Studies using animal models or laboratory methods instead of human subjects.
- Studies focusing on interventions to reduce exposure levels rather than examining dermal and inhalation exposure.
- Case reports, letters, editorials, and reviews without original data.
- Articles for which full-text access was not available.

### Data extraction

After identifying eligible articles, a data extraction form was developed to collect relevant information. This data included the first author, publication year, drugs studied, sampling location, type of chemotherapy, sampled surfaces, surface sample concentration (ng/cm2), air sampling methods, and air sample concentration (ng/m3). Two researchers independently extracted the data using Excel 2013 (Microsoft Office).

### Critical appraisal of studies

The methodological quality of the included studies was assessed using the Joanna Briggs Institute (JBI) Critical Appraisal Tools [14]. Given the cross-sectional design of the studies, the JBI Critical Appraisal Checklist for Analytical Cross-Sectional Studies was employed [15]. This tool evaluates key aspects of study quality, including the clarity of inclusion criteria, the validity and reliability of exposure measurements, the identification and handling of confounding factors, the appropriateness of statistical analyses, and ethical considerations. Each study was independently appraised by two reviewers, with discrepancies resolved through discussion. The results of the critical appraisal informed the overall interpretation of the review's findings, with particular attention given to studies with identified limitations.

## Results

### Study selection

**Fig. 1:**
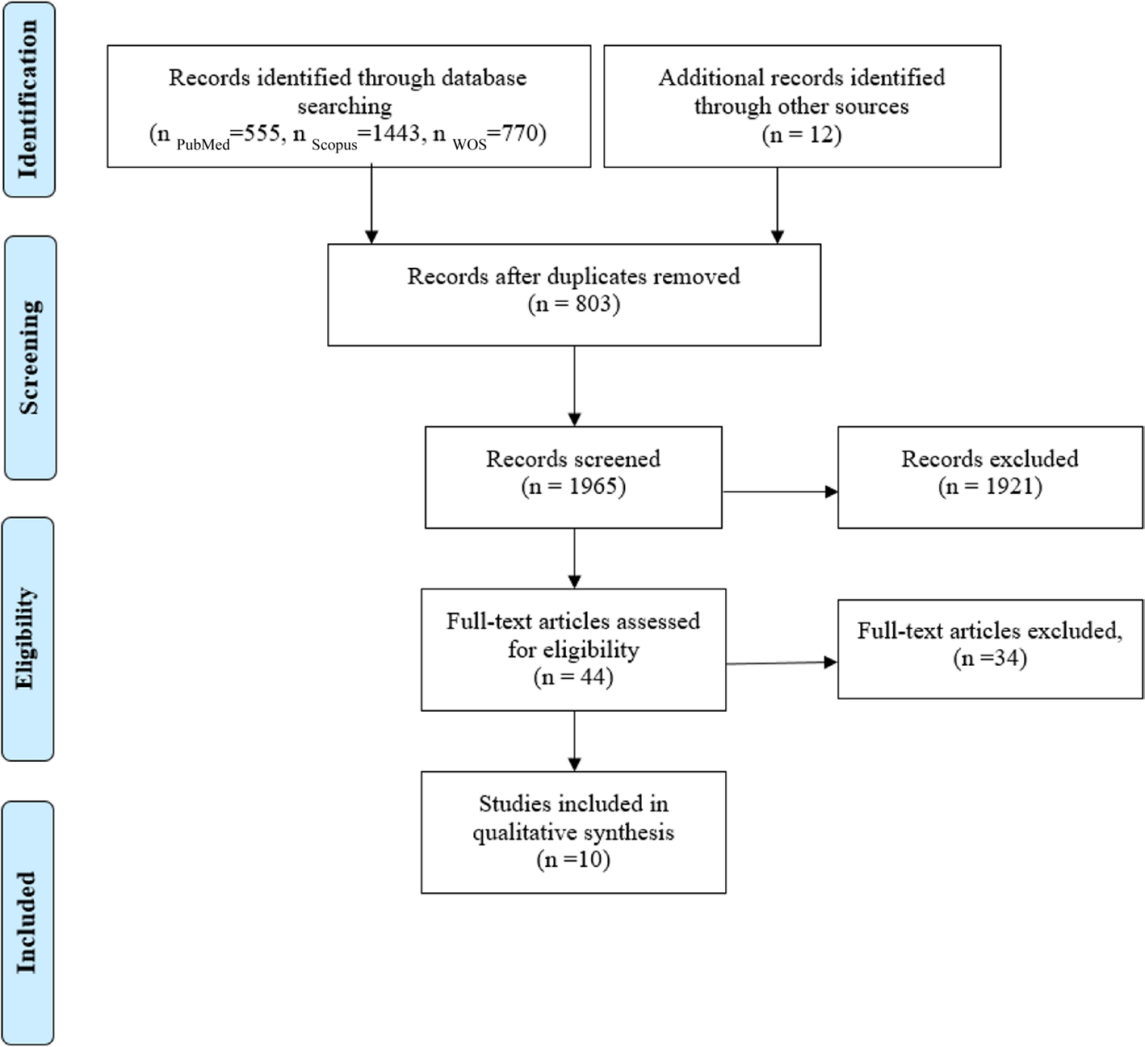
Flow diagram of the screening process and study selection

A search of the EMBASE database via Scopus, MEDLINE via PubMed, and Web of Science in 2023 yielded a total of 2,768 research papers (555 from MEDLINE, 1,443 from EMBASE, and 770 from Web of Science). An additional 12 references were identified through manual searches on Google Scholar and by examining the reference lists of the included studies and relevant reviews. After removing duplicates, 1,965 articles remained for preliminary screening based on their titles and abstracts. Of these, 44 articles met the criteria for a full-text review. After the full-text review, 34 articles were excluded for not meeting the inclusion criteria. Consequently, this systematic review includes only 10 studies [16–25]. The study selection process is depicted in Fig. 1.

### Study characteristics

Ten studies [16–25] were included in this systematic review, focusing on the comparative analysis of dermal and inhalation exposure to various antineoplastic drugs among workers in different workplace settings. Table 1 summarizes the key characteristics of these studies, which were conducted over a range of years and geographic locations.

**Table 1:** Characteristics of the included studies

Number	Author (Year)	Drug Under Investigation	Sampling Location	Type of Chemotropic	Sampled Surfaces	Surface Sample Concentration (ng/cm^2^)	Air Sampling	Air Sample Concentration (ng/m^3^)
1	Jung et al. (2023) [16]	Doxorubicin	Veterinary operating room	PIPAC and RIPAC	PIPAC and RIPAC devices, around the surgical table	< 0.16−181.07	Environmental sampling around the surgical table (near surgeons, nurses, or anesthesiologists), entry doors, or hallways)	ND^*^
2	Roussin et al. (2021) [17]	Cisplatin, Doxorubicin	Operating room	ePIPAC	Various surfaces (floor, injector sheet, cover sheet, perfusion bag) and personal protective equipment	Cisplatin: $<959$ Doxorubicin:$<0.00029$	Environmental sampling (during drug preparation, syringe injection, and air waste management)	ND
3	Ametsbichler et al. (2018) [18]	Cisplatin / Oxaliplatin	operating room	PIPAC	Surgical room (injector, workstation, gloves)	0.00001−1.733	Continuous environmental sampling during PIPAC within 25–50 cm from the surgical table	$<0.0031$
4	Villa et al. (2015) [19]	Oxaliplatin	Operating room	-	Surgical table, various locations in the operating room, oxaliplatin perfusion bags, gloves, shoes of surgeons and nurses	$ND-6.613$	Environmental sampling (above the surgical field, near the oxaliplatin perfusion device, inside and outside the operating room, near the door)	0.18−0.5
5	Sessink et al. (2015) [20]	Cyclophosphamide	Pharmacy with robotic system	-	Surfaces inside and outside the cleanroom. Vials, bags, bag ports, gloves	$\text{ND }- 0.33$	Environmental sampling (in front of CytoCare (CC) and in front of the biological safety cabinets (BSCs)opposite of CC) and personal sampling (the technicians handling CC)	ND
6	Kushnir et al. (2013) [21]	Cyclophosphamide	operating room	-	-	$\text{ND }- 0.8$	Environmental and personal breathing zone sampling area (the inpatient pharmacy during chemotherapy solution preparation; the operating room; before, during, and after the mock procedure; during the cleaning of the operating room, and during the sterilization of the surgical equipment)	ND
7	Gorná et al. (2011) [22]	5-Fluorouracil	Hospital pharmacy and outpatient clinic	-	-	$<0.0000075$	Environmental sampling	$<4.3$
8	Huang et al. (2010) [23]	5-Fluorouracil	pharmacy intravenous admixture service of a hospital	-	the surface of different areas in PIVAS and personal protective equipments	0.11−99.89	Environmental sampling	ND
9	Sessink et al. (1994) [24]	5-Fluorouracil	Pharmaceutical manufacturing plant	-	-	$<1- 630$	-	$75000$
10	Sessink et al. (1992) [25]	Cyclophosphamide, Ifosfamide, 5-Fluorouracil, Methotrexate	Clinical pharmacy, preparation section (Administration), oncology section (Administration)	-	Work trays, floors, entry points, gloves in the preparation room	cyclophosphamide: ND ifosfamide: ND methotrexate: ND fluorouracil:$0 - 0.5$	Environmental sampling (during preparation of the drugs)	ND

^*^ND Not Detectable

This review covers a broad spectrum of antineoplastic drugs across the included studies, reflecting the variety of substances assessed for occupational exposure risks. The drugs investigated include Doxorubicin [16, 17], Cisplatin [18], Oxaliplatin [18, 19], Cyclophosphamide [20, 21], 5-Fluorouracil [22–25], Ifosfamide [25], and Methotrexate [25]. Some studies focused on individual drugs [16, 19–24], such as Doxorubicin and Oxaliplatin, while others examined combinations [17, 18, 25], offering a comprehensive view of the diverse antineoplastic drugs studied within workplace settings. This analysis provides valuable insights into the complexities of drug exposure and helps assess the potential health risks associated with their use in occupational environments.

The systematic review encompasses a diverse array of sampling locations, illustrating the varied settings in which antineoplastic drug exposure is assessed. These locations include veterinary operating rooms [19], conventional operating rooms [20–22, 24], hospital pharmacies with robotic systems [23, 25], and outpatient clinic spaces [25]. Additional settings include pharmacy intravenous admixture services [26] and pharmaceutical manufacturing plants [26]. The studies also examined clinical pharmacy areas [27], specifically the preparation and oncology sections, providing a comprehensive overview of the environments where antineoplastic drug exposure is a critical concern. These diverse sampling locations contribute to understanding the potential risks to workers in various workplace environments and offer insights into the complexities of exposure assessment.

The studies reviewed demonstrated differences in the methodologies used for administering chemotherapy in various workplace settings. In operating rooms, chemotherapy was typically administered using methods such as ePIPAC, PIPAC, and RIPAC [16, 18], designed to ensure precise drug delivery and therapeutic efficacy. In contrast, research in pharmaceutical settings explored a broader range of administration methods. Some studies investigated traditional methods [22, 23, 25], while others focused on advanced robotic systems [20]. The diversity of chemotherapy methodologies underscores the need for a thorough assessment of drug exposure risks and a deep understanding of the unique challenges and safety measures associated with each approach.

### Surface contamination

This systematic review included a detailed examination of surface sampling to evaluate dermal exposure to antineoplastic drugs across various occupational settings. The studies analyzed a wide range of surfaces in healthcare facilities, from those near PIPAC and RIPAC devices to surgical tables in operating rooms [16]. Researchers examined floors, injector components, protective equipment, and biological safety cabinets [17, 23]. Some studies focused on cleanroom interiors, including vials, bags, and gloves [18], while others extended to exterior areas [20]. Notably, some studies did not explicitly mention the surfaces analyzed, indicated by a "-" symbol [21, 22, 24]. The broad range of surfaces investigated highlights the challenges in assessing antineoplastic drug exposure in different work settings.

Quantification of surface sample concentrations across these studies revealed significant variability in antineoplastic drug exposure. For example, Jung et al. [16] reported Doxorubicin contamination ranging from less than 0.16 ng/cm2 to a notable high of 181.07 ng/cm2, while Roussin et al. [17] found significantly lower levels, frequently below 0.00029 ng/cm2. In contrast, Cisplatin concentrations in Roussin et al.’s study [17] reached up to 959 ng/cm2. Other studies on Cisplatin and Oxaliplatin reported concentrations ranging from 0.00001 to 1.733 ng/cm2 [18]. Another study found Oxaliplatin concentrations ranging from "ND" (Not Detectable) to 6.613 ng/cm2 [19]. Cyclophosphamide concentrations varied from "ND" to 0.33 ng/cm2 depending on the workplace setting [20, 21]. One study on 5-Fluorouracil reported surface concentrations as low as 0.0000075 ng/cm2, while another study in a hospital pharmacy with a robotic system revealed a broader range from 0.11 ng/cm2 to 99.89 ng/cm2 [23]. Further research on Cyclophosphamide, Ifosfamide, Methotrexate, and Fluorouracil reported a wide range of results, with some drugs registering as "ND" for surface sample concentrations and others, like Fluorouracil, reporting concentrations up to 630 ng/cm2 [24, 25]. These findings emphasize the complex and variable nature of antineoplastic drug contamination on workplace surfaces, highlighting the need for a thorough systematic review to understand these exposures fully.

### Airborne contamination

This systematic review also examined air sampling methods across the selected studies, providing an overview of the diverse strategies employed to assess antineoplastic drug exposure in occupational settings. Environmental sampling was conducted in various locations, such as around surgical tables, near surgical teams, entryways, and hallways [16]. Continuous environmental sampling within a specific range from the surgical table was used to assess PIPAC and RIPAC exposure, providing insights into drug exposure during critical procedures such as drug preparation, syringe injection, and waste management [17–19].

Some studies [20, 21] combined environmental and personal sampling techniques to gain a more comprehensive understanding of exposure dynamics. These methods were applied in settings including pharmacies during antineoplastic drug preparation and operating rooms before, during, and after surgeries, as well as during cleaning and equipment sterilization processes. While some studies focused solely on general environmental sampling [22, 23, 25], others had a broader scope, examining exposure in multiple settings and areas [16, 17, 19–21]. The variety of air sampling methods underscores the complexities of assessing antineoplastic drug exposure in occupational settings, emphasizing the multifaceted nature of these investigations. This methodological diversity captures the complexities of drug exposure dynamics, contributing to a more complete understanding of the potential risks faced by workers.

The analysis of air sample concentrations in the reviewed studies revealed a wide range of values, reflecting variations in the presence of antineoplastic drugs in workplace air. Most studies [16, 17, 20, 21, 23, 25] reported "ND" (Not Detectable) concentrations, indicating the absence of detectable drugs in the sampled air. However, some studies found low concentrations of drugs, such as less than 0.0031 ng/m3 [18] and 0.18–0.5 ng/m3 [19], indicating measurable but limited drug presence. In one study [24] conducted in a pharmaceutical manufacturing plant, air sample concentrations reached up to 75,000 ng/m3, suggesting a significant risk of exposure for workers. These variations highlight the diverse nature of air sample concentrations and underscore the importance of assessing and understanding the presence of antineoplastic drugs in workplace air, particularly regarding potential health risks for exposed individuals.

### Quality assessment of included studies

The methodological quality of the ten included studies was appraised using the JBI Critical Appraisal Checklist for Analytical Cross-Sectional Studies. The appraisal revealed that 4 studies were of high quality [17–20], 3 were moderate to high [16, 21, 23], and 3 were moderate [22, 24, 25]. Most studies demonstrated robust methodologies for measuring occupational exposures and employed valid statistical analyses. However, several studies [16–18, 20, 23–25] exhibited limitations, particularly in their handling of confounding. Notably, older studies like those by Sessink et al. (1994) [24] and Sessink et al. (1992) [25] had less rigorous reporting standards, especially regarding ethical approvals and the sensitivity of exposure detection methods. These limitations suggest a need for caution when interpreting the results of these older studies. Table 2 provides a summary of the quality assessment for each study.

**Table 2:** Summary of quality assessment for included studies

Study	Quality Assessment	Key Strengths	Key Limitations
Jung et al. (2023) [16]	Moderate to High	Valid exposure measurements, clear inclusion criteria	Lack of detailed discussion on confounding factors
Roussin et al. (2021) [17]	High	Robust methodological approach, reliable measurements	Limited discussion on confounding factors
Ametsbichler et al. (2018) [18]	High	Valid and reliable measurements, appropriate statistical analysis	Minimal elaboration on confounding factors
Villa, A. F., et al. (2015) [19]	High	Detailed setting description, valid outcome measurements	Lack of sophisticated statistical analysis
Sessink et al. (2015) [20]	High	Strong methodology, effective use of robotic systems to reduce exposure	Limited strategies to mitigate confounding factors
Kushnir et al. (2013) [21]	Moderate to High	Adequate measurement methods, clear setting description	Lack of advanced statistical analysis
Gorná et al. (2011) [22]	Moderate	Clear focus on surface contamination, valid measurement techniques	Limited statistical analysis, lack of confounder discussion
Huang et al. (2010) [23]	Moderate to High	Detailed surface contamination analysis, robust statistical significance	Lack of ethical considerations, minimal confounding factor discussion
Sessink et al. (1994) [24]	Moderate	Important historical context, significant findings on airborne contamination	Older methods, lack of discussion on confounders and ethical considerations
Sessink et al. (1992) [25]	Moderate	Valid methods for the time, comprehensive exposure assessment	Lack of confounding factor analysis, outdated methods

## Discussion

### Summary of key findings

This systematic review synthesized evidence from ten studies [16–25] focusing on dermal and inhalation exposure to antineoplastic drugs among workers. The central research question was whether dermal exposure outweighed inhalation exposure, providing insights into the occupational hazards faced by workers in various settings. The key findings of this systematic review are summarized as follows:

- Out of the initial 2,768 research papers identified across three major databases and additional sources, only ten studies [16–25] met the inclusion criteria. This highlights a significant need for more in-depth research in this area.
- The studies covered a wide range of antineoplastic drugs, including Doxorubicin [16, 17], Cisplatin [18], Oxaliplatin [18, 19], Cyclophosphamide [20, 21, 25], 5-Fluorouracil [22–25], Ifosfamide [25], and Methotrexate [25]. This variety is essential for understanding the complexities of drug exposure across different drugs and workplace settings.
- The sampling locations varied widely, covering occupational environments such as veterinary operating rooms [16], conventional operating rooms [17–19, 21], hospital pharmacies with conventional [23, 24] and robotic systems [20], outpatient clinics [22], and pharmaceutical manufacturing plants [25]. The inclusion of a pharmaceutical manufacturing plant [25] is particularly significant, as it represents a markedly different occupational setting compared to healthcare environments. This study reported exceptionally high levels of airborne antineoplastic drug contamination, underscoring the unique exposure risks faced by factory workers. These findings raise concerns about the adequacy of current safety protocols in manufacturing settings and suggest a need for updated practices to ensure better protection for workers.
- Variations in chemotherapy administration methods were observed in both operating rooms [16–19, 21] and pharmaceutical settings [20, 22–25]. This emphasizes the importance of assessing drug exposure risks and understanding the unique challenges and safety measures associated with each approach.
- Surface sampling revealed significant variability in antineoplastic drug exposure. For example, Jung et al. (2023) [16] found Doxorubicin surface contamination ranging from less than 0.16 ng/cm^2^ to 181.07 ng/cm^2^, while Roussin et al. (2021) [17] reported Cisplatin concentrations up to 959 ng/cm^2^. These findings underscore the necessity for targeted safety measures depending on the specific drugs and settings involved.
- The variety of air sampling methods reflected the complexities of assessing antineoplastic drug exposure, as demonstrated by the studies in this review. For instance, while Roussin et al. (2021) [17], and Kushnir et al. (2013) [21] reported "Not Detectable" (ND) levels using environmental sampling around surgical tables and during procedures, Sessink et al. (1994) [24] employed stationary and personal breathing zone sampling in a pharmaceutical manufacturing plant, where they detected exceptionally high concentrations of 5-fluorouracil (up to 75,000 ng/m^3^). This variation underscores the challenges in accurately capturing exposure levels and highlights the importance of tailored sampling strategies to effectively assess and mitigate occupational exposure risks.

### Dermal vs. inhalation exposure

The findings provide valuable insights into the comparative analysis of dermal and inhalation exposure to antineoplastic drugs. Both routes of exposure are significant, but their extent and implications vary. Dermal exposure occurs across different surfaces and sampling locations, with varying contamination levels, emphasizing the need for specific protective measures depending on the drug and workplace.

Inhalation exposure, as indicated by air sampling, presents a broad spectrum of outcomes. For instance, studies by Roussin et al. (2021) [17] and Kushnir et al. (2013) [21] reported 'ND' (Not Detectable) levels of airborne antineoplastic drugs, suggesting minimal inhalation risk in those specific environments. However, the study by Sessink et al. (1994) [24] starkly contrasts these findings, revealing significantly hazardous concentrations of 5-fluorouracil, reaching up to 75,000 ng/m3, in a pharmaceutical manufacturing plant. This highlights the variability and potential severity of inhalation exposure depending on the environment.

Interestingly, while surface sampling across various studies often revealed diverse levels of contamination, such as the 181.07 ng/cm2 of doxorubicin reported by Jung et al. (2023) [16], airborne concentrations in many reviewed studies, including those by Roussin et al. (2021) [17] and Kushnir et al. (2013) [21], frequently remained undetectable. This disparity emphasizes the distinct risks associated with different routes of exposure. Particularly in manufacturing environments, as evidenced by Sessink et al. (1994) [24], airborne contamination can reach alarmingly high levels, posing a significant risk to workers.

The exceptionally high airborne concentrations reported by Sessink et al. (1994) [24] in a pharmaceutical manufacturing plant underscore the critical need to address occupational exposure risks beyond healthcare settings. These findings are especially concerning given the likely less stringent safety practices of the time, pointing to an urgent need for contemporary studies and updated safety protocols in such environments. The 1994 study serves as a reminder of the importance of understanding the dynamics of antineoplastic drug exposure, particularly in settings where airborne contamination can pose serious risks to workers. The wide variability in air sample concentrations across different studies reinforces the need for comprehensive and tailored safety measures to mitigate these risks effectively.

These findings have important implications for workplace safety and health risk assessments. They emphasize the need for accurately determining the primary route of exposure for each antineoplastic drug, as different drugs exhibit varying behaviors in terms of volatilization and surface contamination. A tailored risk assessment approach focusing on the predominant exposure pathway for each specific drug is required.

Furthermore, safety measures must be tailored to the predominant route of exposure. Personal protective equipment (PPE), safe handling procedures, and comprehensive surface decontamination should be prioritized where dermal exposure is common [27, 28]. In contrast, environments with inhalation risks require local exhaust ventilation, continuous air monitoring, and the use of respiratory protection [27, 29].

### Factors influencing exposure

Several factors influence workplace exposure to antineoplastic drugs [30, 31]. One of the most notable findings from our review is the significant variation in exposure levels across different workplace settings. This variability is evident in both dermal and inhalation exposure routes, depending on the specific drug involved. For instance, in terms of dermal exposure, Jung et al. (2023) [16] reported Doxorubicin surface concentrations ranging from less than 0.16 ng/cm2 to as high as 181.07 ng/cm2, while Roussin et al. (2021) [17] found Cisplatin concentrations consistently reaching up to 959 ng/cm2. Additionally, Willaert et al. [32] detected no traces of antineoplastic drugs in air samples collected from the breathing zones of surgeons and anesthesiologists following ePIPAC procedures. In stark contrast, Sessink et al. [24] reported extremely high levels of 5-fluorouracil (75,000 ng/m3) in air samples taken from a pharmaceutical factory. The significant differences in exposure levels observed across the included studies underscore the complexity of this issue and the need for further investigation to understand the factors contributing to such variability.

Several factors may contribute to the observed variations in exposure levels, including but not limited to the following:

#### Workplace practices

The selection of containment systems, safety procedures, cleaning methods, and personal protective equipment (PPE) can vary across settings, influencing the likelihood of drug contamination. For example, in a study conducted by Ametsbichler et al. [18], the presence of cisplatin and oxaliplatin contamination was investigated in two healthcare facilities. When comparing these two hospitals, it was found that all calculated percentiles (including the minimum, 25th, 50th, 75th, and maximum) for Pt concentrations were significantly higher in Hospital A than in Hospital B. These differences were statistically significant (*p* = 0.008). One possible explanation for this difference is that, in Hospital B, the micropump was removed "en bloc" with the trocars at the end of the PIPAC procedure, whereas in Hospital A, it was removed before the trocars. This could explain why Hospital A had higher contamination levels on the head ends of the trocars.

The cleaning method is another protocol that can influence environmental contamination levels. Roussin et al. [17] found residual cisplatin and doxorubicin contamination with maximum concentrations of 9.5 ng/cm2 and 0.27 pg/cm2, respectively, even after cleaning the floor according to a standardized protocol. Cross-contamination occurred between the two PIPACs due to inefficient cleaning. The efficacy of chemical decontamination of chemotherapy work surfaces is determined by the cleaning solution used and the cleaning protocol followed. According to the literature, detergent disinfectants based on quaternary ammonium or surfactants are effective in reducing antineoplastic drug contamination on floors [33, 34]. While the detergent disinfectant product used in this study appears appropriate, the cleaning procedures should be revised to include multiple single-use mops for floor cleaning, as saturation of each mop with antineoplastic drugs can cause contamination of other areas and create a cumulative effect.

#### Drug handling procedures

Differences in exposure can arise from variations in how healthcare professionals handle and administer antineoplastic drugs. Contamination levels may be influenced by factors such as the method of administration, frequency of drug preparation, and adherence to safety guidelines [35, 36]. For instance, technical or human errors can occur during the use of ePIPAC equipment or trocars, including issues such as disconnecting high-pressure lines, primary leakage, spillage during ventilation, and errors during the connection, disconnection, or removal of the syringe. The amount of drug used is another factor that can influence contamination levels. In the study by Roussin et al. (2021) [17], higher levels of surface contamination with cisplatin were observed, likely due to the use of five times more cisplatin.

#### Pharmaceutical formulations

This refers to the drug’s form of administration, such as powder, liquid, capsule, or prefilled syringe. The formulation of a drug, along with its physical and chemical properties, is a crucial factor in determining the precautions needed to prevent exposure. For example, powders require different risk management strategies compared to prefilled syringes [1, 37]. Despite the recognized significance of these differences, none of the included studies specifically addressed the impact of various pharmaceutical formulations of antineoplastic drugs on surface contamination.

#### Drug characteristics

The physicochemical properties of specific drugs can influence their tendency for contamination and aerosolization. Factors such as solubility, volatility, and the presence of active pharmaceutical ingredients can significantly impact exposure levels [37]. For example, at 25 °C, 5-Fluorouracil has a vapor pressure of approximately 2.7 × 10⁻⁶ mmHg [38], whereas Oxaliplatin has a vapor pressure of approximately 6.78 × 10⁻1⁰ mmHg [19]. These figures indicate that Oxaliplatin has a significantly lower vapor pressure than 5-Fluorouracil. Consequently, 5-Fluorouracil is more prone to evaporation than Oxaliplatin, which could explain the higher level of airborne contamination observed with 5-Fluorouracil.

#### Equipment and infrastructure

Differences in equipment and infrastructure may also contribute to exposure disparities. For example, the maximum drug concentration on surfaces in pharmacies with robotic systems has been reported as 0.33 ng/cm2 [20], while in conventional pharmacies, this value has been reported as high as 99.89 ng/cm2 [23]. Furthermore, the Pressurized Intraperitoneal Aerosol Chemotherapy (PIPAC) concept is a relatively new therapeutic approach, which may lead to hazardous exposure situations during various procedural steps [18]. Studies by Jung et al. [16], Roussin et al. [17], and Ametsbichler et al. [18] identified technical or human errors involving PIPAC equipment or trocars, such as disconnection of the micropump or high-pressure lines, primary leakage, spillage during ventilation, connection or disconnection of the syringe, and operative complications requiring medical or technical intervention. These issues can result in significant surface contamination.

Hyperthermic Intraperitoneal Chemotherapy (HIPEC) is another new treatment approach used to treat peritoneal carcinomatosis. During the initial surgical phase, electrocautery generates a large amount of smoke containing steam, particulate matter, organic and inorganic substances, and microorganisms. Villa et al. [19] used the open-abdomen technique in their study, raising concerns about anticancer drug exposure among healthcare workers during open-abdomen HIPEC surgery, especially since most operating room personnel have limited experience with these drugs. Before administration, antineoplastic drugs are heated to facilitate vaporization. The open-abdomen technique requires manual control of the chemotherapy solution distribution within the abdomen, which increases the risks of splashes and direct contamination of the surgeon.

In the study, no significant oxaliplatin contamination was found in the atmosphere during the HIPEC procedure. However, heavy oxaliplatin contamination was observed on the operating table and floor near the surgeon's feet. These contaminations were most likely caused by spills and splashes during the surgeon's manual supervision of intra-abdominal oxaliplatin perfusion. As a result, the surgeon's overshoes (or shoes if overshoes were not used) became contaminated. Slight contamination of the shoes underneath the overshoes was also detected in surgeons wearing overshoes, and residual contamination on the floor at the surgeon's feet was observed before HIPEC, indicating that standard cleaning procedures may not be entirely effective.

#### Sampling locations

The choice of sampling locations can significantly impact the observed exposure levels. Various studies have focused on specific areas within the workplace, highlighting variations across surfaces and environments. For example, Jung et al. [16] investigated air and surface contamination resulting from PIPAC and RIPAC procedures performed on a pig model. The results showed that doxorubicin surface contamination was detected in 5 out of 51 surface samples collected by wiping. These contaminated samples were primarily obtained from devices that had been directly exposed to aerosols within the abdominal cavity. Following PIPAC and RIPAC procedures, specific equipment, such as the telescope, trocar, and syringe line connector, exhibited contamination. Because the telescope and trocar were introduced into the abdominal cavity, they presented a higher risk of surface contamination due to antineoplastic drug aerosolization. Additionally, a drug leak caused by droplet spillage from an incomplete connection of the doxorubicin-containing syringe to the spray nozzle resulted in a contamination level of 181.07 ng/cm2.

#### Sensitivity of detection methods

Variability in detection methods, sensitivity, and thresholds may lead to discrepancies in reported drug concentrations. Less sensitive analytical techniques may fail to detect lower levels of contamination that more sensitive methods can identify [39]. Various methods were employed in the included studies to detect and quantify specific drugs and their related substances. For example, Jung et al. [16] used ultra-high performance liquid chromatography-tandem mass spectrometry to detect Doxorubicin in air and on surfaces, with limits of detection (LODs) of 14.4 ng/m3 for air samples and 0.16 ng/cm2 for surface samples. Roussin et al. [17] used inductively coupled plasma-mass spectrometry to detect Cisplatin and liquid chromatography-tandem mass spectrometry to detect Doxorubicin, with LODs of 10 ng/wipe and 5 ng/filter for Cisplatin and 5 pg/wipe for Doxorubicin. Ametsbichler et al. [18] employed inverse voltammetry to measure total platinum (Pt) concentrations, with LODs of 0.02 ng/wipe sample and 6 pg/air sample. Villa et al. [19] measured Pt concentrations using inductively coupled plasma mass spectrometry, with LODs of 0.03 ng/filter and 0.2 to 0.5 ng/m3 for air concentrations and 0.25 ng/wipe or 0.27 pg/cm2 for surface concentrations. These differences in sensitivity may explain why pollutants are not detected in some areas.

#### Sampling frequency and duration

The frequency and duration of environmental sampling can significantly impact contamination detection. Short-term increases in exposure may be captured by more frequent or prolonged sampling periods [40]. Among the studies reviewed, the largest sampled air volume was 1.95 cubic meters in the study by Ametsbichler et al. [18], which detected 0.0031 ng/m3 of platinum. Gorná et al. (2011) [22] collected 0.33–0.84 L of air, which contained 4.3 ng/m3 of 5-fluorouracil. In contrast, Jung et al. [16] sampled 1 cubic meter of air but found no airborne pollutants.

### Health implications

The variability in contamination levels in both surface and air sampling, such as the high levels of 5-fluorouracil found by Sessink et al. (1994) [24] compared to the 'ND' levels reported in other studies, underscores the potential health risks that workers face, depending on the specific environment and the antineoplastic drugs they are exposed to. For example, Sessink et al. (1994) [24]reported an exceptionally high 5-fluorouracil concentration of up to 75,000 ng/m3 in the air at a pharmaceutical manufacturing plant, indicating a significant inhalation risk. Conversely, studies like Roussin et al. (2021) [17] found 'ND' levels in air samples despite high surface contamination, suggesting that while some exposures may be minor, others—especially in high-concentration scenarios—can pose serious health risks. The potential health consequences include both acute and chronic effects, with even low levels of these hazardous drugs posing risks over time [41, 42].

Biological monitoring of occupational exposure is a critical tool for assessing and managing antineoplastic drug-related chemical risks [43]. It is the most effective method for assessing internal contamination because it considers all exposure pathways (respiratory, dermal, oral), the use of personal protective equipment (PPE) or lack thereof, the effectiveness of PPE, personal hygiene practices, work practices, and the amount of antineoplastic drugs handled at the individual level. In the included studies, there was no evidence of internal contamination after working with antineoplastic drugs [44]. The findings demonstrated the effectiveness of the individual and collective protective measures used in these studies [17, 19, 20, 45].

### Implications for occupational safety

The findings of this systematic review have significant implications for occupational safety in environments where antineoplastic drugs are handled. By understanding the complexities of both dermal and inhalation exposures, as well as the variability in contamination levels observed across different settings, this review highlights the need for evidence-based guidelines and targeted exposure-reduction strategies. Notably, the study conducted in a pharmaceutical manufacturing plant [27] reveals a critical gap in our current understanding of exposure risks outside of healthcare settings. The high levels of contamination reported in this study underscore the urgent need to develop specific safety guidelines tailored to industrial environments where antineoplastic drugs are manufactured. These findings stress the importance of expanding occupational health research to include factory settings, which present unique challenges and risks compared to those in healthcare environments. To effectively safeguard workers, occupational safety measures must be tailored to account for the specific drugs involved, the conditions of the workplace, and the appropriate protective measures required for each unique setting.

To address the implications of our findings, we must consider the safeguards that facilities and professionals in various occupational settings should implement. Contamination of reusable devices and surfaces in occupational settings puts staff at risk. Injectors are frequently stored in areas where staff may come into contact with them without proper protection, as observed in the studies. Ametsbichler et al. [18] recommend using single-use transparent films to shield PIPAC injectors as a protective measure. Remarkably, our review uncovered an interesting trend where contamination on injector surfaces was higher before PIPAC procedures compared to afterward. This phenomenon, also observed in other contexts such as HIPEC procedures, is likely due to insufficient cleaning following the previous procedure, inadvertently leading to contamination spreading to other surfaces. In terms of the floor, there was clearly insufficient cleaning and cross-contamination. In one case, despite cleaning between two subsequent PIPACs, Ametsbichler et al. [18] found high Pt residues of 172 pg/cm2 (out of 480 pg/cm2 before cleaning) on the floor next to the operating table and 104 pg/cm2 on the nearby area under the injector. Effective protection strategies are critical in various occupational settings where contaminated surfaces and devices are a reality.

Overshoes are required for surgeons in practice; shoes worn underneath overshoes must not be personal shoes, but rather work shoes (disposable shoes or shoes that can undergo a decontamination procedure). The efficacy and acceptability of available floor protection devices should be tested to ensure they do not limit the surgeons' movements or increase the risk of slipping. The level of contamination on the floor indicates that cleaning staff are also significantly exposed to anticancer drugs. As a result, they should wear gloves and overshoes when cleaning the operating room, and their actual exposure should be assessed more precisely. The low level of external contamination observed in studies [18] of oxaliplatin perfusion bags is consistent with that previously reported in hospital pharmacies preparing anticancer drugs and justifies the systematic use of gloves when handling these items.

The included study [19] found that the outer gloves were heavily contaminated, the second set of gloves was slightly contaminated, and the surgeon's hands were uncontaminated. Based on our findings, surgeons should be advised to use three sets of gloves when administering perioperative intraperitoneal chemotherapy via the open-abdomen procedure. Because surgical gloves do not completely prevent anticancer drug penetration during prolonged contact [46], it is also recommended to change gloves every 30 min when working with antineoplastic drugs, as well as after obvious contamination. Surgeons who work with antineoplastic drugs should also wear outer gloves that cover the elbow [47]. Due to the possibility of cross-contamination, surgeons and nurses should thoroughly wash their hands before leaving the operating room. To prevent secondary contamination, protective barrier garments such as gloves, gowns, pajamas, overshoes, and shoes that may have been contaminated with anticancer drugs should be placed in dedicated containers within the operating room. Gloves, overshoes, and surgical gowns should be discarded, while pajamas and shoes can be decontaminated in a separate container.

Finally, our systematic review, guided by numerous studies, sheds light on the complex landscape of antineoplastic drug exposure in occupational settings. This understanding compels us to prioritize the safety of professionals and workers in their critical roles while administering these life-saving drugs. Understanding the health risks and implementing appropriate protective measures are critical steps in ensuring the safety of those who work with these hazardous substances.

### Study quality and implications for findings

The critical appraisal of the included studies revealed a generally moderate to high level of methodological quality, with notable variability among the studies. High-quality studies, such as those by Jung et al. (2023) [16] and Roussin et al. (2021) [17], employed comprehensive methodologies and rigorous reporting standards. These studies provided robust evidence of occupational exposure risks, particularly through their detailed examination of exposure routes, meticulous data collection, and advanced statistical analyses. These strengths contribute to the reliability of their findings and reinforce the importance of stringent safety measures in workplaces where antineoplastic drugs are handled.

In contrast, some of the older studies, such as those by Sessink et al. (1994) [24] and Sessink et al. (1992) [25], exhibited methodological limitations, particularly in ethical considerations and the management of confounding factors. During the period when these studies were conducted, the focus on these aspects was less rigorous, potentially influencing the study outcomes. For instance, the lack of detailed confounder analysis and the absence of clear ethical reporting may have introduced biases that could affect the validity of their conclusions. Therefore, while these studies provide valuable historical context, their findings should be interpreted with caution, especially when compared to more recent research that adheres to higher methodological standards.

The variability in study quality across the included research underscores the need for continuous improvement in this field. Future studies should prioritize the thorough identification and control of confounding factors, as this is crucial for drawing accurate conclusions about occupational exposure risks. Moreover, enhancing the sensitivity and specificity of exposure measurement techniques will improve the detection and quantification of antineoplastic drugs in various occupational settings. Adhering to strict ethical guidelines is also essential to protect study participants and ensure the credibility of the research.

Furthermore, the review highlights several areas where methodological improvements are necessary. For example, older studies often lacked comprehensive reporting on the sensitivity of their detection methods, which could lead to underestimation of exposure levels. This limitation points to the need for standardized protocols that can be consistently applied across studies to facilitate more accurate comparisons and meta-analyses.

The evidence from high-quality studies underscores the importance of addressing the identified gaps in the research. By focusing on methodological rigor and ethical standards, future research can strengthen the evidence base, leading to more definitive conclusions regarding occupational exposure to antineoplastic drugs. This, in turn, will support the development of evidence-based guidelines and safety protocols that better protect workers in healthcare and industrial settings.

Overall, this systematic review provides a comprehensive assessment of the current state of research on occupational exposure to antineoplastic drugs, highlighting both the strengths and weaknesses of the existing literature. The findings underscore the critical need for ongoing research that not only fills the gaps identified in older studies but also builds on the strengths of more recent investigations. By improving the methodological rigor and addressing the limitations noted in this review, future studies can contribute to a safer working environment for those who are regularly exposed to these hazardous substances.

## Limitations

While this systematic review provides valuable insights into antineoplastic drug exposure in the workplace, several limitations must be acknowledged. There is a potential for publication bias, as studies with significant findings are more likely to be published, which could affect the comprehensiveness of the evidence. Additionally, the diverse methodologies employed across the included studies introduce methodological heterogeneity, making direct comparisons and broad conclusions challenging. Geographic and temporal variations further complicate the findings, as differences in regulations and safety measures across regions and timeframes may influence exposure levels. Moreover, population heterogeneity, with varying worker practices and safety protocols, may contribute to inconsistencies in exposure levels. Lastly, some studies lacked comprehensive data reporting, particularly regarding surface and air sample analyses, which could impact the precision of the conclusions. These limitations highlight the need for cautious interpretation of the results and underscore the importance of future research to address these gaps and strengthen the evidence base for occupational health in the context of antineoplastic drug exposure.

## Recommendations for future research

The gaps identified in this systematic review highlight several areas for future research. Notably, there is a pressing need for more studies on the exposure of factory workers involved in the production of antineoplastic drugs. Findings from Sessink et al. (1994) [24] suggest that these workers may be exposed to significantly higher levels of airborne contaminants, revealing both a gap in our understanding and potential deficiencies in current protective measures. To ensure comprehensive occupational safety across all environments where antineoplastic drugs are handled, future research should prioritize investigating and mitigating these risks. Additionally, conducting more comprehensive and standardized studies across various occupational settings is crucial for enhancing our understanding of antineoplastic drug exposure. Future research should also focus on the long-term health effects of such exposures and the development of effective interventions to reduce them.

## Conclusion

This systematic review provides a comprehensive examination of dermal and inhalation exposure to antineoplastic drugs in various occupational settings. The review highlights significant variability in contamination levels, emphasizing the need for safety measures tailored to the predominant exposure route for each drug.

Key findings underscore the importance of accurate risk assessments, standardized safety protocols, and the use of sensitive detection methods to protect workers from hazardous drug exposure. Even low levels of exposure can pose both acute and chronic health risks, making biological monitoring and preventive strategies essential in all settings where these drugs are handled.

The review also acknowledges limitations, including potential publication bias, variations in data quality, and population heterogeneity, which should be considered when interpreting the findings.

Overall, this review serves as a valuable resource in enhancing the safety of those handling antineoplastic drugs. Future research should focus on addressing the identified gaps, particularly by conducting more comprehensive studies on long-term health effects and developing effective interventions to reduce exposure.

## Data Availability

The datasets generated and/or analyzed during the current investigation are available upon reasonable request from the corresponding authors. We are committed to promoting transparency and collaboration in research, and we welcome inquiries regarding our data to facilitate further understanding and discussion in the scientific community. Please contact: Seyed Jamaleddin Shahtaheri at shahtaheri@tums.ac.ir for access to the relevant datasets.

## References

[1] Gruenewald B, Gilkey D. Hazardous drug exposure in healthcare. World Safety J. 2021;30:72–6.

[2] Beigzadeh Z, Golbabaei F, Khadem M, Shahtaheri SJ. Fabrication and optimization of molecularly imprinted nanofibers in assessment of occupational exposure to 5-fluorouracil. J Mazandaran Univ Med Sci. 2019;29(179):49–64.

[3] (IARC) IAfRoC. IARC monographs on the evaluation of carcinogenic risks to humans: pharmaceutical drugs. 2012.

[4] Beigzadeh Z, Golbabaei F, Khadem M, Omidi F, Someah MS, Shahtaheri SJ. Development of Molecularly Imprinted Membranes for Selective Determination of Urinary Ultra-Trace 5-Fluorouracil as Antineoplastic Drug Used in Chemotherapy. Macromol Res. 2020;28(4):390–9.

[5] Mozaffari S, Bayatian M, Hsieh N-H, Khadem M, Garmaroudi AA, Ashrafi K, Shahtaheri SJ. Reconstruction of exposure to methylene diphenyl-4, 4′-diisocyanate (MDI) aerosol using computational fluid dynamics, physiologically based toxicokinetics and statistical modeling. Inhalation Toxicol. 2023;35:1–15. 10.1080/08958378.2023.2285772 38019695

[6] Connor TH, DeBord G, Pretty JR, Oliver MS, Roth TS, Lees PSJ, Krieg EF, Rogers B, Escalante CP, Toennis CA, et al. Evaluation of Antineoplastic Drug Exposure of Health Care Workers at Three University-Based US Cancer Centers. J Occup Environ Med. 2010;52(10):1019–27. 20881620 10.1097/JOM.0b013e3181f72b63

[7] Beigzadeh Z, Golbabaei F, Khadem M, Pourhassan B, Pourbabaki R, Kalantari S, Shahtaheri SJ. Synthesis and Optimization of a Molecularly Imprinted Membrane as a Specific Absorbent to Assess the Occupational Exposure to the 5-Fluorouracil Drug. In J Nanosci. 2021;20(1):2150003.

[8] Fransman W, Vermeulen R, Kromhout H. Dermal exposure to cyclophosphamide in hospitals during preparation, nursing and cleaning activities. Int Arch Occup Environ Health. 2005;78(5):403–12. 15887018 10.1007/s00420-004-0595-1

[9] Aliabadi MM, Naderi G, Shahtaheri SJ, Forushani AR, Mohammadfam I, Jahangiri M. Mechanical and barrier properties of XNBR-clay nanocomposite: a promising material for protective gloves. Iran Polym J. 2014;23:289–96.

[10] Hedmer M, Tinnerberg H, Axmon A, Jönsson BAG. Environmental and biological monitoring of antineoplastic drugs in four workplaces in a Swedish hospital. Int Arch Occup Environ Health. 2008;81(7):899–911. 18066576 10.1007/s00420-007-0284-y

[11] Borm PJ, Kreyling W. Toxicological hazards of inhaled nanoparticles—potential implications for drug delivery. J Nanosci Nanotechnol. 2004;4(5):521–31. 15503438 10.1166/jnn.2004.081

[12] Henderson LK, Craig JC, Willis NS, Tovey D, Webster AC. How to write a Cochrane systematic review. Nephrology. 2010;15(6):617–24. 20883282 10.1111/j.1440-1797.2010.01380.x

[13] Parums DV. Review articles, systematic reviews, meta-analysis, and the updated preferred reporting items for systematic reviews and meta-analyses (PRISMA) 2020 guidelines. Med Sci Monit. 2021;27:e934475–e934471. 34421116 10.12659/MSM.934475 PMC8394590

[14] Robin K, Buccheri RN. CSB, MLIS: Critical Appraisal Tools and Reporting Guidelines for Evidence-Based Practice. Worldviews Evid Based Nurs. 2017;14(6):463–72. 28898556 10.1111/wvn.12258

[15] Porritt K, Judith G, Lockwood C. JBI’s systematic reviews: study selection and critical appraisal. Am J Nurs. 2014;114(6):47–52. 24869584 10.1097/01.NAJ.0000450430.97383.64

[16] Jung W, Park M, Park SJ, Lee EJ, Kim HS, Chung SH, Yoon C. Occupational Exposure during Intraperitoneal Pressurized Aerosol Chemotherapy Using Doxorubicin in a Pig Model. Saf Health Work. 2023;14(2):237–42. 37389318 10.1016/j.shaw.2023.04.002 PMC10300457

[17] Roussin F, Taibi A, Canal-Raffin M, Cantournet L, Durand-Fontanier S, Druet-Cabanac M, El Balkhi S, Maillan G. Assessment of workplace environmental contamination and occupational exposure to cisplatin and doxorubicin aerosols during electrostatic pressurized intraperitoneal aerosol chemotherapy. Eur J Surg Oncol. 2021;47(11):2939–47. 34034944 10.1016/j.ejso.2021.05.020

[18] Ametsbichler P, Böhlandt A, Nowak D, Schierl R. Occupational exposure to cisplatin/oxaliplatin during Pressurized Intraperitoneal Aerosol Chemotherapy (PIPAC)? Eur J Surg Oncol. 2018;44(11):1793–9. 29871821 10.1016/j.ejso.2018.05.020

[19] Villa AF, El Balkhi S, Aboura R, Sageot H, Hasni-Pichard H, Pocard M, Elias D, Joly N, Payen D, Blot F, et al. Evaluation of oxaliplatin exposure of healthcare workers during heated intraperitoneal perioperative chemotherapy (HIPEC). Ind Health. 2015;53(1):28–37. 25327298 10.2486/indhealth.2014-0025 PMC4331192

[20] Sessink PJM, Leclercq GM, Wouters DM, Halbardier L, Hammad C, Kassoul N. Environmental contamination, product contamination and workers exposure using a robotic system for antineoplastic drug preparation. J Oncol Pharm Pract. 2015;21(2):118–27. 24567041 10.1177/1078155214522840

[21] Kushnir CL, Fleury AC, Couch J, Hill MC, Spirtos NM. Evaluation of exposures to healthcare personnel from cisplatin during a mock demonstration of intra-operative intraperitoneal chemotherapy administration. Gynecol Oncol. 2013;130(2):350–3. 23648469 10.1016/j.ygyno.2013.04.467

[22] Gorná L, Odráska P, Dolezalová L, Piler P, Oravec M, Bláha L. Determination of airborne and surface contamination with cyclophosphamide at the Masaryk Memorial Cancer Institute, Brno, Czech Republic. Ceska Slov Farm. 2011;60(1):25–31. 21650015

[23] Huang YW, Zhang NH, Tong DM, Feng X, Zhang MB, He JL. Investigation on occupational exposure to 5-fluorouracil in pharmacy intravenous admixture service of a hospital. Zhonghua Lao Dong Wei Sheng Zhi Ye Bing Za Zhi. 2010;28(6):414–7. 21033150

[24] Sessink PJM, Timmersmans JL, Anzion RBM, Bos RP. Assessment of occupational exposure of pharmaceutical plant workers to 5-fluorouracil determination of alpha-fluoro-beta-alanine in urine. J Occup Environ Med. 1994;36(1):79–83. 8138854

[25] Sessink PJ, Boer KA, Scheefhals AP, Anzion RB, Bos RP. Occupational exposure to antineoplastic agents at several departments in a hospital. Environmental contamination and excretion of cyclophosphamide and ifosfamide in urine of exposed workers. Int Arch Occup Environ Health. 1992;64(2):105–12. 1399019 10.1007/BF00381477

[26] Brun G. Formulation of aqueous core capsules for the triggered release of proteins (Doctoral dissertation, Université Pierre et Marie Curie-Paris VI); 2015.

[27] Power LA, Coyne JW, Hawkins B. ASHP guidelines on handling hazardous drugs. Am J Health Syst Pharm. 2018;75(24):1996–2031. 30327293 10.2146/ajhp180564

[28] Connor TH, McDiarmid MA. Preventing occupational exposures to antineoplastic drugs in health care settings. CA Cancer J Clin. 2006;56(6):354–65. 17135692 10.3322/canjclin.56.6.354

[29] Power LA, Coyne JW. ASHP guidelines on handling hazardous drugs. Am J Health Syst Pharm. 2018;75(24):1996–2031. 30327293 10.2146/ajhp180564

[30] Srisintorn W, Geater A, Polovich M, Thongsuksai P. Factors influencing precautions against antineoplastic drug exposure among nurses and nurse assistants in Thailand. Int Arch Occup Environ Health. 2021;94(5):813–22. 33427994 10.1007/s00420-020-01649-9

[31] Meijster T, Fransman W, Van Hemmen J, Kromhout H, Heederik D, Tielemans E. A probabilistic assessment of the impact of interventions on oncology nurses’ exposure to antineoplastic agents. Occup Environ Med. 2006;63(8):530–7. 16551759 10.1136/oem.2005.022723 PMC2078132

[32] Willaert W, Sessink P, Ceelen W. Occupational safety of pressurized intraperitoneal aerosol chemotherapy (PIPAC). Pleura and Peritoneum. 2017;2(3):121–8. 30911641 10.1515/pp-2017-0018 PMC6328076

[33] Adé A, Chauchat L, Frève JFO, Gagné S, Caron N, Bussières JF. Comparison of decontamination efficacy of cleaning solutions on a biological safety cabinet workbench contaminated by cyclophosphamide. Can J Hosp Pharm. 2017;70(6):407–14. 29298999 10.4212/cjhp.v70i6.1708 PMC5737182

[34] Queruau Lamerie T, Nussbaumer S, Décaudin B, Fleury-Souverain S, Goossens J-F, Bonnabry P, Odou P. Evaluation of decontamination efficacy of cleaning solutions on stainless steel and glass surfaces contaminated by 10 antineoplastic agents. Ann Occup Hyg. 2013;57(4):456–69. 23223271 10.1093/annhyg/mes087

[35] Fazel SS, Keefe A, Shareef A, Palmer AL, Brenner DR, Nakashima L, Koehoorn MW, McLeod CB, Hall AL, Peters CE. Barriers and facilitators for the safe handling of antineoplastic drugs. J Oncol Pharm Pract. 2022;28(8):1709–21. 34612752 10.1177/10781552211040176

[36] Simegn W, Dagnew B, Dagne H. Knowledge and associated factors towards cytotoxic drug handling among University of Gondar Comprehensive Specialized Hospital health professionals, institutional-based cross-sectional study. Environ Health Preventive Med. 2020;25(1):11. 10.1186/s12199-020-00850-z PMC7155262 32284041

[37] Hazardous drug exposures in healthcare. https://www.cdc.gov/niosh/topics/hazdrug/riskmanagement.html.

[38] Compound Summary: Fluorouracil. https://pubchem.ncbi.nlm.nih.gov/compound/Fluorouracil.

[39] Guichard N, Guillarme D, Bonnabry P, Fleury-Souverain S. Antineoplastic drugs and their analysis: a state of the art review. Analyst. 2017;142(13):2273–321. 28560370 10.1039/c7an00367f

[40] Sbarra AN, Harris AD, Johnson JK, Madger LS, O’Hara LM, Jackson SS, Thom KA. Guidance on frequency and location of environmental sampling for Acinetobacter baumannii. Infect Control Hosp Epidemiol. 2018;39(3):339–42. 29378673 10.1017/ice.2017.319 PMC6342278

[41] Lombardo J, Roussel C. Highlighting the risk of occupational exposure to hazardous drugs in the health care setting. Pharm Times. 2018;15.

[42] Ivanova K, Avota M. Antineoplastic Drugs: Occupational Exposure and Side Effects. Proc Latv Acad Sci Sect B. 2016;70(5):325–9.

[43] Leso V, Sottani C, Santocono C, Russo F, Grignani E, Iavicoli I. Exposure to Antineoplastic Drugs in Occupational Settings: A Systematic Review of Biological Monitoring Data. Int J Environ Res Public Health. 2022;19(6):3737. 35329423 10.3390/ijerph19063737 PMC8952240

[44] Manno M, Viau C, Cocker J, Colosio C, Lowry L, Mutti A, Nordberg M, Wang S. Biomonitoring for occupational health risk assessment (BOHRA). Toxicol Lett. 2010;192(1):3–16. 19446015 10.1016/j.toxlet.2009.05.001

[45] Sessink PJM, Vandekerkhof MCA, Anzion RBM, Noordhoek J, Bos RP. Environmental contamination and assessment of exposure to antineoplastic agents by determination of cyclophosphamide in urine of exposed pharmacy technicians - is skin absorption an important exposure route. Arch Environ Health. 1994;49(3):165–9. 8185386 10.1080/00039896.1994.9940377

[46] Stuart OA, Stephens AD, Welch L, Sugarbaker PH. Safety monitoring of the coliseum technique for heated intraoperative intraperitoneal chemotherapy with mitomycin C. Ann Surg Oncol. 2002;9:186–91. 11888877 10.1007/BF02557372

[47] González-Bayón L, González-Moreno S, Ortega-Pérez G. Safety considerations for operating room personnel during hyperthermic intraoperative intraperitoneal chemotherapy perfusion. Eur J Surg Oncol. 2006;32(6):619–24. 16672186 10.1016/j.ejso.2006.03.019

